# Overlapping Community Compositions of Gut and Fecal Microbiomes in Lab-Reared and Field-Collected German Cockroaches

**DOI:** 10.1128/AEM.01037-18

**Published:** 2018-08-17

**Authors:** Madhavi L. Kakumanu, Julia M. Maritz, Jane M. Carlton, Coby Schal

**Affiliations:** a Department of Entomology and Plant Pathology and Center for Human Health and the Environment, North Carolina State University, Raleigh, North Carolina, USA; b Center for Genomics and Systems Biology, Department of Biology, New York University, New York, New York, USA; University of Georgia

**Keywords:** German cockroach, *Blattella* microbiota, microbiome, gut, feces, fecal microbiota, indoor microbiome

## Abstract

German cockroaches, Blattella germanica (Blattodea: Ectobiidae), are human commensals that move freely between food and waste, disseminating bacteria, including potential pathogens, through their feces. However, the relationship between the microbial communities of the cockroach gut and feces is poorly understood. We analyzed the V4 region of the 16S rRNA gene and the V9 region of the 18S rRNA gene by next-generation sequencing (NGS) to compare the bacterial and protist diversities in guts versus feces and males versus females, as well as assess variation across cockroach populations. Cockroaches harbored a diverse array of bacteria, and 80 to 90% of the operational taxonomic units (OTUs) were shared between the feces and gut. Lab-reared and field-collected cockroaches had distinct microbiota, and whereas lab-reared cockroaches had relatively conserved communities, considerable variation was observed in the microbial community composition of cockroaches collected in different apartments. Nonetheless, cockroaches from all locations shared some core bacterial taxa. The eukaryotic community in the feces of field-collected cockroaches was found to be more diverse than that in lab-reared cockroaches. These results demonstrate that cockroaches disseminate their gut microbiome in their feces, and they underscore the important contribution of the cockroach fecal microbiome to the microbial diversity of cockroach-infested homes.

**IMPORTANCE** The German cockroach infests diverse human-built structures, including homes and hospitals. It produces potent allergens that trigger asthma and disseminates opportunistic pathogens in its feces. A comprehensive understanding of gut and fecal microbial communities of cockroaches is essential not only to understand their contribution to the biology of the cockroach, but also for exploring their clinical relevance. In this study, we compare the diversity of bacteria and eukaryotes in the cockroach gut and feces and assess the variation in the gut microbiota across cockroach populations.

## Abstract (precis)

The German cockroach infests diverse human-built structures, including homes and hospitals. It produces potent allergens that trigger asthma and disseminates opportunistic pathogens in its feces. A comprehensive understanding of gut and fecal microbial communities of cockroaches is essential not only to understand their contribution to the biology of the cockroach, but also for exploring their clinical relevance. In this study, we compare the diversity of bacteria and eukaryotes in the cockroach gut and feces and assess the variation in the gut microbiota across cockroach populations.

## INTRODUCTION

Cockroaches (Blattodea) are a diverse group of insects that inhabit a wide range of habitats, including deserts, tropical rainforests, temperate habitats, and specialized commensal habitats, such as ant and termite nests and human-built structures (1, 2). The cockroach microbiome comprises horizontally transmitted microbes and nematodes and vertically transmitted symbionts. Most cockroach species have symbiotic associations with transovarially transmitted intracellular bacteria, Blattabacterium spp., that inhabit specialized cells in the fat body (3) and are involved in uric acid metabolism, amino acid production, and nitrogen recycling (4–7).

The cockroach gut harbors a wide variety of microorganisms, some of which collaborate in digestion, including that of lignocellulosic compounds, and act as barriers against pathogen colonization (8–10). Distinct bacterial assemblages inhabit the three sections of the alimentary canal of cockroaches, and the hindgut in particular is colonized by a highly abundant and diverse community of bacteria (10–14) comprising opportunistic, facultative, and commensal microbiota (15). Recent high-throughput sequencing studies have demonstrated diverse gut microbiota associated with several cockroach species representing divergent taxa (9, 11, 16–19).

The gut microbiome of cockroaches is influenced by various exogenous factors (e.g., diet and the local habitat) and endogenous factors (e.g., gut pH). The gut bacterial load of the German cockroach, Blattella germanica (Ectobiidae), was shown to double from the first to the second nymphal stage and stabilize thereafter, while the microbial compositions differed significantly between adults and nymphs (9). The gut bacterial composition of B. germanica was also shown to be highly dynamic and varied with diet (20), and higher gut bacterial diversity is often reported in wild cockroaches than in lab colonies (16, 17, 20). While few studies have examined physicochemical factors of the gut as potential determinants of the gut community in cockroaches, several such studies have been reported in termites (21–23). Similarly, the presence of some microbes and competition for nutrients might determine the colonization success of certain groups influencing gut community composition.

Cellulolytic protists are also associated with the digestive system in the wood-feeding cockroach Cryptocercus punctulatus (Cryptocercidae) and the lower termites (24, 25). The protists are themselves colonized by nitrogen-fixing ectosymbionts, which might be necessary in the lignin-rich environment of the hindgut (25–27), suggesting a critical role played by flagellates in wood digestion. However, the flagellate diversity associated with the gut of omnivorous cockroaches, like B. germanica, has not been explored.

The mechanisms of acquiring a stable gut microbial community are poorly understood in cockroaches. Coprophagy, which is common in a wide range of insects, including cockroaches (28), might be an important mechanism of horizontal transmission of gut microbes. In B. germanica, neonate cockroaches acquire essential bacteria by coprophagy (10, 29), as well as nutritional benefits (30). Nevertheless, the relationship between the gut and feces microbiomes remains uninvestigated.

The German cockroach is the most common indoor pest in human residences, food processing and storage sites, hospitals, and livestock facilities (31). Research over the last 2 decades has shown that the German cockroach produces an array of allergens that can cause allergies and trigger asthma (32–35). Cockroach movement between the kitchen, food materials, and garbage facilitates the translocation of various microbes on their surface, gut, and feces (36–39). A wide range of potentially pathogenic bacteria has been isolated from cockroaches collected in hospitals and livestock operations (38, 40), and some were shown to act as reservoirs of bacteria carrying antibiotic resistance genes (41, 42). Cockroaches also play an important role in the dissemination of cysts of several infectious protozoan parasites (43–45).

Detailed analyses are lacking of the bacterial and protozoan inhabitants of field populations of B. germanica and how their gut and fecal bacterial communities are related. Our goal was to understand how microbial diversity varies between male and female cockroaches, between field-collected and long-term lab-reared cockroaches, and between the cockroach gut and fecal microbiota.

## RESULTS

### Sequences of the V4 hypervariable region of the 16S rRNA gene.

We sequenced a total of 105 samples of B. germanica comprising 30 whole-insect, 33 whole-gut, and fecal samples (generated from 33 individual lab-reared and field-collected adult males and female cockroaches) and 9 carcass samples from lab-reared cockroaches (see Table S1 in the supplemental material). The carcass represents all the remaining tissues after the whole gut was removed. Since our initial sequencing showed that Blattabacterium spp. were the predominant representative in carcass samples (>99%), we did not sequence the carcasses of field-collected cockroaches. After quality check, 27,372,807 high-quality sequences were retained, and samples that had fewer than 50,000 reads were excluded from further analysis. A total of 101 samples (28 whole-insect, 33 whole-gut, 32 fecal, and 8 carcass samples) were used for downstream analysis and taxonomic assignments. A range of 59,173 to 596,608 sequences (mean ± standard deviation [SD], 271,017 ± 64,962) were obtained per sample and binned to 6,425 OTUs at a 97% threshold (Table S3). Samples were rarefied to 50,000 reads/sample for analyzing diversity indices (Fig. S1).

**FIG 1:**
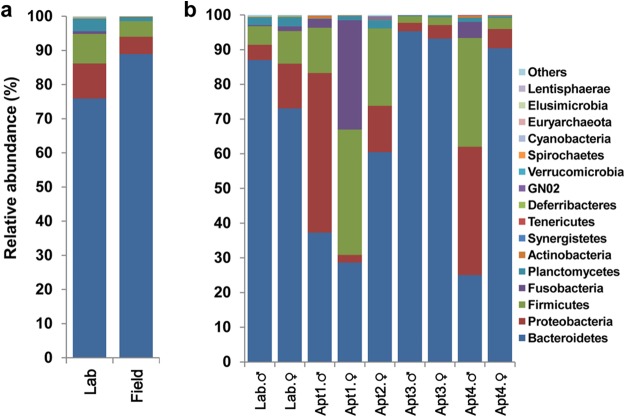
Relative abundance of bacterial phyla, including Blattabacterium spp., in whole-insect samples. (a) Lab-reared and field-collected German cockroaches. Each bar depicts the mean relative abundance value of independent replicates (lab = 8, field = 20). (b) Male and female lab-reared and field-collected B. germanica cockroaches presented by location (Apt, apartment). Bars depict the mean relative abundance values of independent replicates (for Lab, male [M] = 4, female [F] = 4; for Apt1, M = 3, F = 3; for Apt2, F = 3; for Apt3, M = 2, F = 3; for Apt4, M = 3, F = 3).

The overall mean microbial compositions of all the replicates of lab-reared and field-collected cockroaches were remarkably similar at the phylum level, dominated by Bacteroidetes, Firmicutes, and Proteobacteria (Fig. 1a). However, considerable variation in microbial compositions was observed among samples by location (Fig. 1b and S2) and sex and among individual cockroaches from the same location (Fig. S2).

### Taxonomic assignments of bacteria from whole cockroaches. (i) Lab-reared cockroaches.

**FIG 2:**
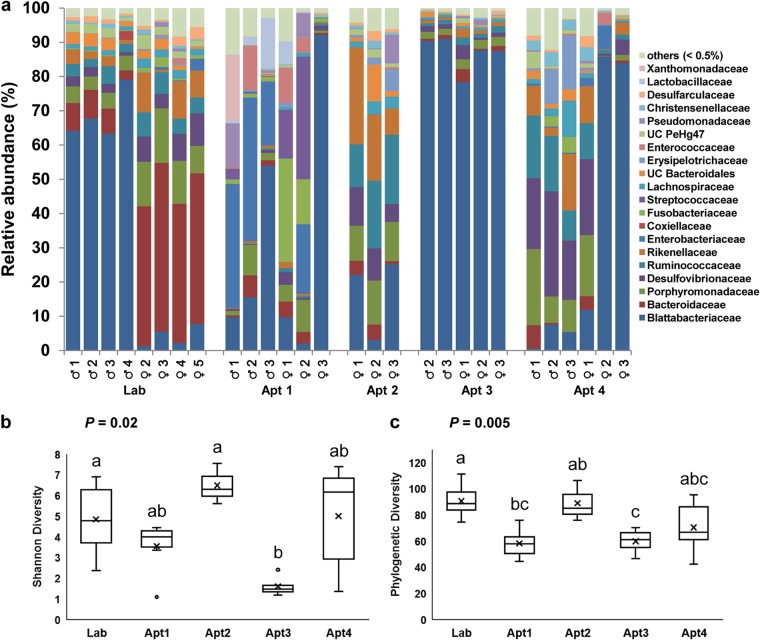
(a) The distribution of bacterial taxa at the family level in whole-body samples of male and female B. germanica cockroaches from lab-reared and field-collected cockroaches. Data include Blattabacterium species. Box plots show the alpha diversities of (b) Shannon diversity indices and (c) Phylogenetic diversity among the whole-insect samples by location. (b and c) Bars delineate the median, and “x” defines the mean, the hinges represent the lower and upper quartiles, the whiskers extend to the most extreme values (no more than 1.5 times the interquartile range from the box), and outliers are plotted as circles, if present. Sample sizes for whole-insects are Lab = 8, Apt1 = 6, Apt2 = 3, Apt3 = 5, Apt4 = 6. UC, unclassified. In panels b and c, different lowercase letters over the bars indicate statistically significant differences between treatments (Kruskal-Wallis test, *P* < 0.05)

The three most abundant bacterial phyla in lab-reared male and female cockroaches were Bacteroidetes (87.0 versus 73.1%, respectively), Proteobacteria (4.4 versus 12.9%, respectively), and Firmicutes (5.3 versus 9.4%, respectively), comprising approximately 95% of the rarefied 16S rRNA gene sequences (Fig. 1b). Other phyla, including Planctomycetes, Deferribacteres, Elusimicrobia, Spirochaetes, Synergistetes, Tenericutes, and Verrucomicrobia, were present in relatively low abundances. A significant difference (adonis, *R*^2^ = 0.64, *P* < 0.001) was observed in the microbial composition of lab males and females (Fig. 2a and S3a and b). Although members of Blattabacterium, a known endosymbiont of cockroaches, were detected in all the samples, their relative abundances differed significantly between the sexes in lab-reared cockroaches (males, 63.4 to 79.1%; females, 1.3 to 7.8%). Members of families Blattabacteriaceae, Bacteroidaceae, Porphyromonadaceae, Rikenellaceae, Ruminococcaceae, Enterobacteriaceae, and Desulfovibrionaceae were commonly found in both males and females, but we observed significant differences in the relative abundances of Blattabacteriaceae (males, 71.2%; females, 4.3%; *t* test, *P* < 0.001) and Bacteroidaceae (6.5% versus 46.5%; *t* test *P* < 0.001) between the sexes (Fig. 2a and S3a and c).

### (ii) Apartment-collected cockroaches.

Although there was inherent variation among B. germanica cockroaches collected from different apartments, their microbial diversity was stable at the phylum level and similar to that of lab-reared cockroaches (Fig. 1a and b and S2). However, the relative abundances of bacterial taxa at lower taxonomic levels were significantly different among apartments (adonis, *R*^2^ = 0.37, *P* < 0.01) but not between the sexes (adonis, *R*^2^ = 0.04, *P* = 0.44) (Fig. 2a, S3a, and S4). Although cockroaches from the same apartment shared greater similarity in their microbial community composition than cockroaches from different apartments, a few notable exceptions were observed in the microbial representation in males and females from the same location (Fig. 2a). For instance, Enterobacteriaceae were more abundant in males from apartment 1 (Apt1), whereas Fusobacteria (31%) and Streptococcaceae (25%) dominated the microbial community of females in this apartment. Likewise, Desulfovibrionaceae constituted 25% of the total reads in males in Apt4 but only 4.5% in females. These differences, though substantial, were not statistically significant, likely because of the inherent variation among apartments and small sample size. The relative abundances of Blattabacterium spp. also varied between locations, as they were predominant in Apt3 and Apt4 females, representing approximately 90% of the reads, but their abundances in other samples ranged from 8 to 32% (Fig. 2a and S3d). Notably, however, obvious similarities in the bacterial structures of males and females were observed when the Blattabacterium OTUs were removed.

**FIG 3:**
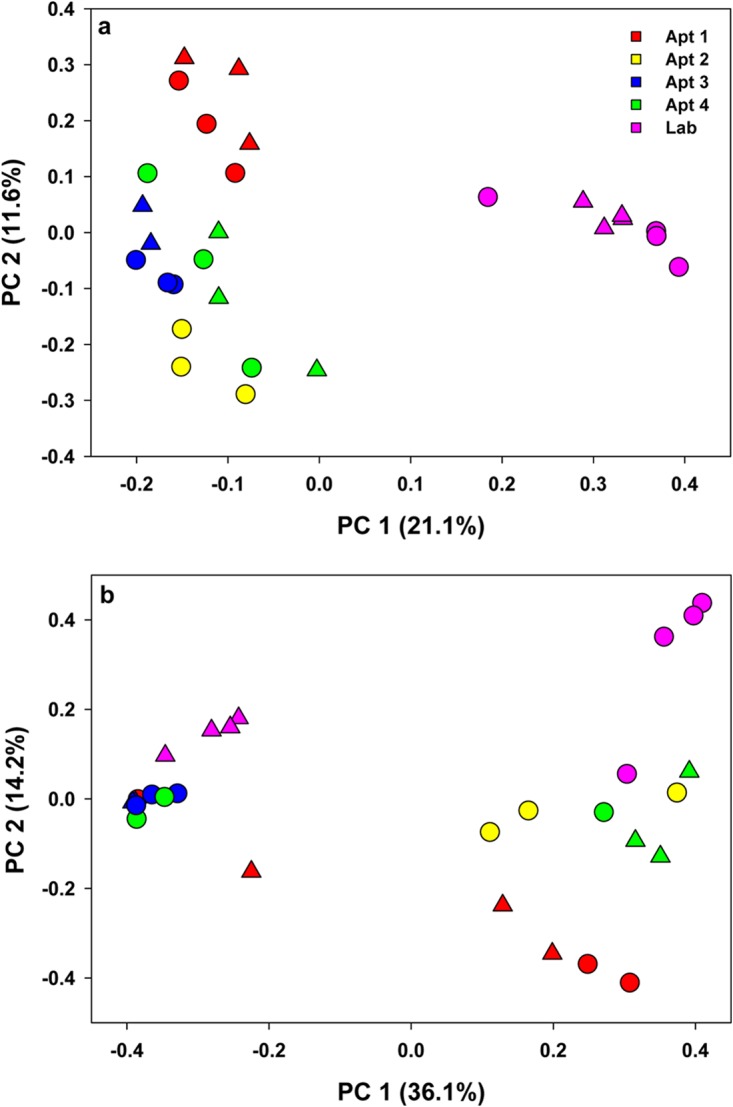
Principal-coordinate analysis depicting differences in taxonomic compositions of bacterial communities among independent replicates of lab-reared and field-collected whole German cockroach samples (males, triangles; females, circles). Community composition dissimilarity is based on the unweighted UniFrac (a) and Bray-Curtis dissimilarity (b) metrics. The percent variation explained by each component is indicated on the axis.

We observed significant variation in the alpha-diversity indices (Shannon and phylogenetic diversity) of samples collected from different locations (Kruskal-Wallis test, *P* < 0.05) (Fig. 2b and c). The unweighted UniFrac analysis showed clear differences in the beta diversity of lab-reared and field-collected cockroaches (Fig. 3a), whereas the Bray-Curtis metrics revealed different clustering patterns between the samples (Fig. 3b), mostly driven by Blattabacterium abundance. However, both the Bray-Curtis and unweighted UniFrac metrics indicated significant differences in the microbial composition of B. germanica by location (adonis, *R*^2^ = 0.37, *P* < 0.001 and *R*^2^ = 0.40, *P* < 0.001, respectively).

### Bacterial taxa in carcass samples.

**FIG 4:**
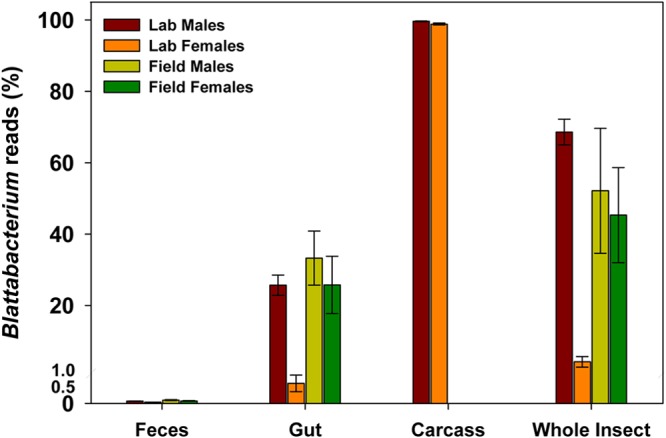
Blattabacterium reads in various tissue samples obtained from lab-reared and field-collected male and female German cockroaches. Error bars represent the standard error of the mean (SEM).

Carcass samples were sequenced only for lab-reared cockroaches, and Blattabacterium spp. represented ≥98% of the sequences in both males and females (Fig. 4). These results confirm observations from previous studies that Blattabacterium spp. inhabit tissues outside the gut mainly in the fat body and ovaries (46).

### Comparison of fecal and gut bacterial taxa.

Blattabacterium reads comprised <0.04% of the total reads in the feces of lab-reared and field-collected cockroaches but were more abundant in gut samples (Fig. 4). Since Blattabacterium is known to be housed in mycetocytes in the fat body (46), it is likely that we did not completely remove fat body tissue while dissecting the gut. Given our objective to compare the bacterial diversity of feces and gut, and since we could not be sure that Blattabacterium spp. were indeed associated with the gut, we bioinformatically removed the Blattabacterium OTUs from the gut and fecal samples of lab-reared and field-collected cockroaches before any further comparisons were made.

**FIG 5:**
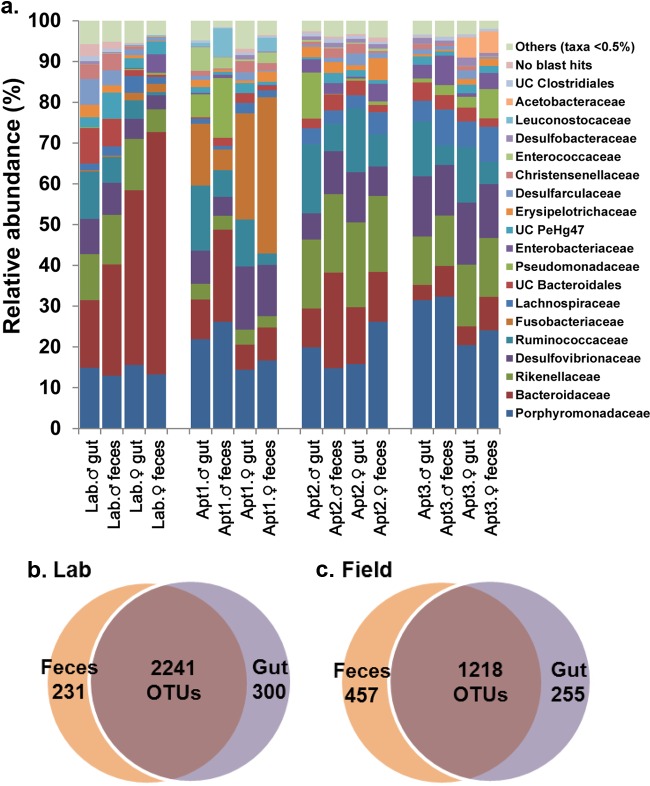
(a) Bar chart depicting the distribution of bacterial taxa at the family level in whole-gut and fecal matter samples of male and female B. germanica cockroaches from lab-reared and field-collected samples presented by location. Blattabacterium was excluded from this analysis. Each bar depicts the mean relative abundance value of independent replicates (Lab male, gut [G] = 4, fecal matter [FM] = 4; Lab female, G = 5, FM = 5; Apt1 male, G = 4, FM = 3; Apt1 female, G = 4, FM = 4; Apt2 male, G = 4, FM = 4; Apt2 female, G = 4, FM = 4; Apt3 male, G = 4, FM = 4; Apt1 female, G = 4, FM = 4). (b and c) Venn diagrams representing the shared OTUs between gut and feces in lab-reared (b) and field-collected (c) B. germanica adults.

**FIG 6:**
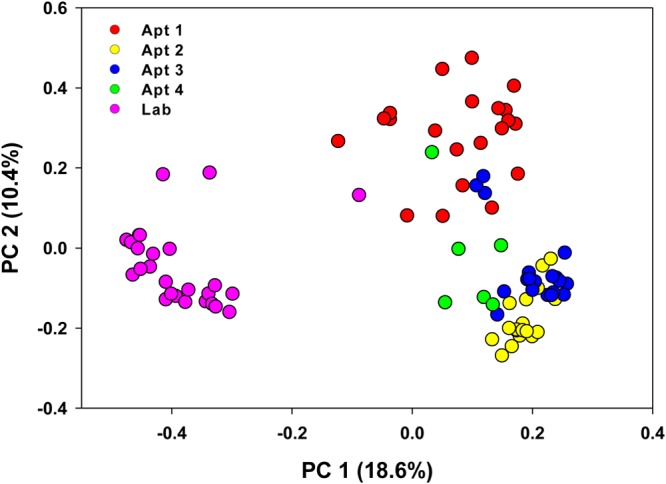
Principal-coordinate analysis depicting differences in taxonomic compositions of bacterial communities among independent replicates of lab-reared and field-collected whole German cockroaches in gut and fecal samples after removing Blattabacterium reads. Community composition dissimilarity is based on the Bray-Curtis dissimilarity metric. The percent variation explained by each component is indicated on the axis.

Despite the individual-level variation, the microbial communities in gut samples broadly overlapped with the respective fecal samples from the same location, sharing about 80 to 90% of the OTUs (adonis, *R*^2^ = 0.02, *P* = 0.23) (Fig. 5a to c, S5, and S6 and Table S5). Across locations, however, we observed significant differences between gut and fecal taxa (adonis, *R*^2^ = 0.38, *P* < 0.001). Nevertheless, a greater similarity was observed between the microbial communities of whole gut and feces that originated from the same cockroach (Fig. S6), and this trend was observed in both lab-reared and field-collected cockroaches. After we removed Blattabacterium from the analysis, the clustering of all the samples, including whole-insect, whole-gut, and fecal samples of both lab-reared and field-collected cockroaches was strongly driven by location (adonis; *R*^2^ = 0.33, *P* < 0.001) (Fig. 6).

### Core bacterial taxa of the German cockroach.

**FIG 7:**
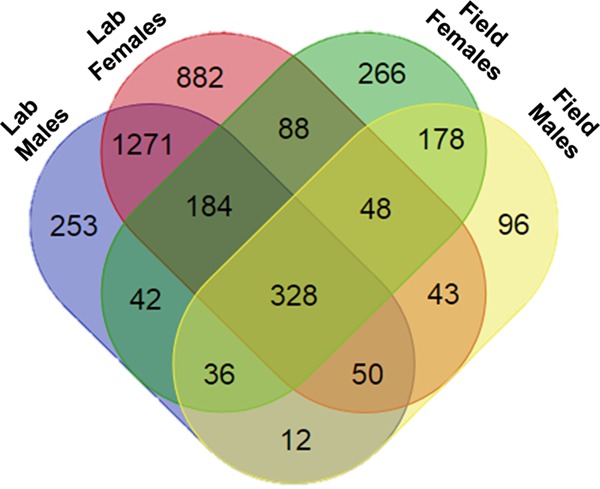
Venn diagrams representing the number of shared OTUs between lab-reared and field-collected B. germanica adults. Sample sizes for lab were male = 4, female = 4; for the field, male = 8, female = 12.

A Venn diagram of all the lab-reared and field-collected cockroaches revealed 328 OTU that were shared among males and females (Fig. 7). The OTUs belonging to Rikenellaceae, Porphyromonadaceae, Lachnospiraceae, Desulfovibrionaceae, and an unclassified family of class VadinHA49 were found in 100% of the gut samples (Table S6). In addition, about 30 other OTUs belonging to the families Coriobacteriaceae, Bacteroidaceae, Pseudomonadaceae, Ruminococcaceae, Enterococcaceae, Christensenellaceae, Enterobacteriaceae, and Desulfarculaceae were present in 95% of the samples irrespective of location and sex, suggesting that these probably comprise the core bacteria of the German cockroach.

### Quantification of 16S rRNA and Blattabacterium.

We conducted quantitative PCR (qPCR) assays to quantify the absolute abundances of total bacteria and Blattabacterium spp. in whole-insect, whole-gut, fecal, and carcass samples. Since we observed substantial variation in the relative abundances of Blattabacterium spp. among samples in our NGS data, we used Blattabacterium as a biomarker to understand the variation. The total bacterial and Blattabacterium abundances were quantified by amplifying the 16S rRNA and penicillin-binding protein (PBP) genes of Blattabacterium, respectively. Similar to NGS, we observed greater variation in the abundance of Blattabacterium spp. among the samples, and overall, a significant positive correlation (Pearson *R* = 0.755, *P* < 0.001) was observed between the qPCR and NGS data (Fig. S7a). However, there was nearly an order of magnitude difference in the 16S rRNA gene and PBP genes of carcass and positive controls (ootheca of B. germanica) (Fig. S7b). We do not know if the difference is because of presence of other bacteria or due to differences in the amplification of the two genes. Thus, the efficiency of the primers and the differential amplification of these two genes need to be tested further.

### Sequences of the V9 hypervariable region of the 18S rRNA gene.

A total of 63 samples (29 fecal, 29 whole gut, and 5 whole insect) were amplified and sequenced. No samples of carcass tissue were attempted, since we expected that the majority of the sequences would be host DNA. After removal of non-18S rRNA gene sequences and data filtering, 2,619,060 high-quality sequences representing 531 OTU and 39 samples (19 fecal, 19 whole gut, and 1 whole insect) remained for downstream analysis. A range of 5,387 to 280,371 (mean ± SD, 67,155 ± 66,389) sequences were obtained per sample. Four of the five whole-insect samples and eight of the nine gut-fecal sample pairs that were sequenced from lab-reared cockroaches contained >99% host DNA, and after removal of these, data from only three of the lab-reared cockroaches remained for downstream analysis.

### Diversity and taxonomy of the eukaryotic community associated with the German cockroach.

**FIG 8:**
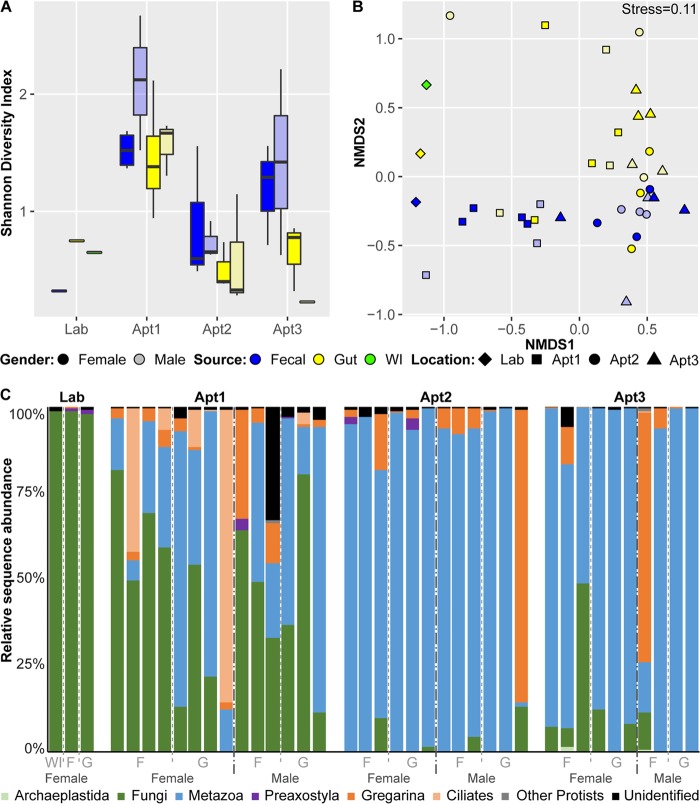
The eukaryotic component of the cockroach microbiome among female and male whole-insect, whole-gut, and fecal samples of B. germanica by location. WI, whole insect; F, feces; G, gut. (a) Alpha-diversity boxplots using the Shannon index. For each group, the bars delineate the median, the hinges represent the lower and upper quartiles, the whiskers extend to the most extreme values (no more than 1.5 times the interquartile range from the box), and outliers are plotted in circles, if present. (b) Nonmetric multidimensional scaling (NMDS) using the Bray-Curtis dissimilarity. (c) Relative abundances of eukaryotic taxa.

Overall, the tissue source of samples (feces, whole gut, or whole insect) was the major driver of eukaryotic diversity and community structure. Higher alpha diversity was observed in the fecal samples than in gut samples and in field-collected samples than in lab-reared samples; however, these differences were not statistically significant (Fig. 8a). Significant variation in alpha diversity was observed between samples collected from different locations (Kruskal-Wallis test, *P* < 0.05). Beta-diversity analysis revealed collection location and tissue source as the major explanatory variables of the eukaryotic community (adonis, *R*^2^ = 0.16 and 0.14, respectively, *P* < 0.001), with distinct clustering patterns between tissue sources (Fig. 8b). No clear clustering was observed between different field locations; however, the adonis test showed these sample groupings to be significant. The environment from which the samples were collected (lab versus field) was also a significant source of variation (adonis, *R*^2^ = 0.093, *P* < 0.001). No significant patterns were found by sex in either of the diversity analyses.

Overall, the eukaryotic cockroach community was dominated by metazoans and fungi (Fig. 8c). The amount of metazoan DNA detected ranged from <1% to 99% and consisted mostly of nematode taxa. Nematodes were detected at high relative abundances (>1%) only in field-collected samples and were primarily represented by the genus Goezia (class Chromadorea). Fungi (Candida species) were also present in high relative abundances among samples, albeit with a broad range (<1% to 98%), and the highest were found in the lab-reared cockroaches. We also detected several taxa known to inhabit the guts of various invertebrate species, including gregarines (phylum Apicomplexa), oxymonads (class Preaxostyla = Anaeromonadea, Excavata), ciliates (order Clevelandellida), and the spore-forming protist Nephridiophaga blattellae (family Nephridiophagidae). These groups were frequently present at low relative abundances (<1%) but showed much more variability across samples, particularly between cockroaches collected from different homes. All of these taxa had higher relative abundances in the fecal samples of field-collected cockroaches, and N. blattellae was not detected at all in samples from lab-reared cockroaches.

Multivariate analysis was performed with MaAsLin, which controls for multiple covariates using a generalized linear model to identify differentially abundant taxa across field-collected cockroaches. Gut samples were enriched for ciliates and negatively associated with gregarines compared to fecal samples (Table S7). Cockroaches collected from Apt2 and Apt3 were enriched for nematodes (Goezia) and depleted for Candida species compared to cockroaches collected from Apt1.

## DISCUSSION

Several key questions of biological and clinical relevance motivated our investigation. (i) What microbes occur in the feces of German cockroaches and do feces accurately represent the gut microbiome? (ii) Do long-term lab-reared cockroaches share similar microbiomes with field-collected cockroaches? Finally, (iii) are there differences in the gut microbiomes of cockroaches collected in various homes? Therefore, we characterized the microbiomes from feces, whole gut, and the carcass (whole body excluding the gut) of males and females from various locations and also from whole insects from the same locations. The results indicated that the microbes that colonize B. germanica cockroaches are influenced by location, and most of the gut bacterial taxa are detected in the feces.

### Blattella germanica microbiome.

The bacterial communities of both lab-reared and field-collected B. germanica cockroaches were dominated by Bacteroidetes, Firmicutes, and Proteobacteria and are consistent with other culture-independent studies of this species (9, 20). The bacterial diversity was similar to that of omnivorous animals and other cockroach species, including the cosmopolitan humidity-adapted Periplaneta americana, xeric-adapted Shelfordella lateralis (Turkestan cockroach), tropical Panchlora sp., and the litter-feeding parthenogenetic Pycnoscelus surinamensis (11, 14, 17–19). We also detected members of other phyla, including Planctomycetes, Deferribacteres, Elusimicrobia, Spirochetes, Synergistetes, Tenericutes, and Verrucomicrobia, as previously reported in B. germanica (9, 20).

At the phylum level, the bacterial community composition was relatively consistent across lab-reared and field-collected B. germanica, and this observation was similar to that of Tinker and Ottesen (17), who observed relatively stable communities in wild-caught and lab-reared P. americana cockroaches. A substantial variation across locations at lower taxonomic levels (family and genus), however, highlights that B. germanica has a greater plasticity in its affiliation with its gut microbial community, unlike the closely related termites (10, 28, 47, 48). We suspect that these differences are related to the omnivorous diet of B. germanica in contrast to the highly restricted and specialized diet of termites.

Despite the pronounced differences between individuals, about 328 OTU were found in nearly all the replicate samples of lab-reared and field-collected cockroaches, probably constituting the core gut bacterial taxa of B. germanica (Fig. 7). The core bacteria constitute a large fraction of the sequences from whole-gut and fecal samples, suggesting their probable involvement in digestion and basic metabolic activities that provide functional stability and gut homeostasis in cockroaches (17, 49). Some of these core bacteria are known to predominate along different sections of the gut. For instance, Lachnospiraceae are associated with the midgut and Ruminococcaceae with the distal hind gut of termites and cockroach species feeding on wood and high-fiber diets (19, 21, 50), and the latter are essential in the digestion of complex carbohydrates. Several of these families are commonly reported in insects, and some families (e.g., Porphyromonadaceae and Rikenellaceae) are associated with healthy gut and in the protection of the host from natural enemies (51). Again, these results are consistent with previous investigations of the gut microbiota of B. germanica (20) and the cockroaches S. lateralis (14, 48), P. americana (16, 17), Panchlora sp. (11), and P. surinamensis (18). The presence of several common OTUs might be an indication of a stable microbial community across B. germanica populations. Blattabacterium spp., the primary endosymbiont of cockroaches, were detected in all the whole-insect samples, and their relative abundances varied across locations and between males and females. Blattabacterium is transovarially transmitted and is considered a nutritional mutualist for its role in nitrogen recycling in cockroaches (5, 6, 52, 53). Thus, the differences in its relative abundance may be related to the nutrition or physiological state of its host, such as vitellogenesis and egg production in females (50). This variation was also reflected in the qPCR assays using Blattabacterium-specific primers. Further investigation is needed to understand the factors that influence the dynamics in the abundance of Blattabacterium in the cockroach host.

### Sources of variation in the Blattella germanica microbiome.

We observed considerable variation in the bacterial community structure among individual cockroaches from the same population, between male and female cockroaches, as well as between field-collected and lab-reared individuals (Fig. 2). The variation in field-collected B. germanica cockroaches is likely related to their omnivorous feeding habit, potentially consuming a wide variety of food sources in different apartments, in contrast to lab-reared insects that have fed on the same diet for hundreds of generations. Changes in the relative proportions of bacterial taxa with diet may be either a reflection of the microbial community in their local environment or adaptive responses of the gut microbial community to specific diets (54–56). Diet is considered a strong factor in shaping the structure of the gut microbiome in various animals, including humans (57–60). In cockroaches, the effects of diet on the gut microbial community have varied significantly across studies, even within the same cockroach species. Thus, significant shifts in gut microorganisms were reported in P. americana (61), whereas Tinker and Ottesen (17) found a stable hindgut microbiome in this species when fed different diets. In the closely related species S. lateralis (also family Blattidae), there was also little evidence of diet-specific microbes, although the gut microbiomes on different diets could readily be separated by nonmetric multidimensional scaling (NMDS) (50). Likewise, the gut microbiome of P. surinamensis fed fungus cultivated by fungus-farming termites was relatively resilient to dietary shifts (18). The importance of time since diet switching on the gut microbiota was illustrated in B. germanica cockroaches, where the bacterial community in females 10 days after switching was generally more diverse and showed more variation than 5 days after diet switching (20).

Most studies of insect gut microbiomes have reported relatively large interindividual variation even among members of the same population, sex, and developmental stage (50, 62). Our results are consistent with this pattern and reinforce the idea that specialized diets and ecological specialization (e.g., termites and wood feeding cockroaches) and the predominance of parent-to-offspring vertical and horizontal transmission of microbes promote a narrow and more consistent microbiota. Conversely, omnivorous insects with a broad ecological niche, and particularly synanthropic cockroaches, likely incorporate environmental microbes into their parent-provisioned microbiota, resulting in a more diverse and variable gut microbiome. Also, we cannot ignore the possible influence of intraspecific genetic variation of the host, physiological state, interactions with numerous biotic and abiotic stressors within a given environment (51), and the presence of gut parasites (63) in shaping the microbial community of the host and the variations at the individual level.

### Gut and fecal microbiomes.

Ordinations of the gut and fecal samples, excluding Blattabacterium, clustered by location and by individual cockroaches (Fig. 6). The gut microorganisms of cockroaches are involved in the digestion and metabolism of food materials and the production of volatile fatty acids and other metabolites that modulate development, nutritional status, and communication of their host (54, 56, 64, 65). These bacteria represent an assemblage of environmental microbes, some of which are associated with food, and core bacteria that are essential and adaptive to the host. Fecal pellets are thus a source of a mutualistic and resident hindgut fauna, free-living “transient” microbes, and both host and microbial metabolites that are beneficial to the newly hatched nymphs (28). The highly overlapping bacterial diversity that we observed in the gut and feces of individual cockroaches is consistent with the idea that because feces serves to horizontally transfer and inoculate nymphs with a mutualistic hindgut microfauna, the gut microbiome is conserved in the feces.

### Eukaryotic diversity of the cockroach microbiome.

Whole-insect samples from lab-reared cockroaches contained significant amounts of host tissue, and therefore after sequencing, bioinformatic removal of these sequences resulted in only three samples containing sufficient data for downstream analysis. Greater sequencing depth in future studies will be needed for adequate coverage after the removal of host OTUs and to test for differences in diversity between lab-reared and field-collected cockroaches. Nonetheless, higher levels of diversity were observed in the feces than the whole guts of field-collected cockroaches, and these samples formed distinct clusters in the NMDS analysis. Thus, hindgut contents need to be dissected and extracted free of cockroach tissue in order to properly sample gut eukaryotes.

The majority of the eukaryotes found in cockroaches were species of fungi or nematodes; however, there was high variability in the relative abundances of these taxa between samples. It is unclear if these taxa represent potential colonizers of the cockroach gut or come from dietary or environmental sources. Further work will be needed to determine their sources and what, if any, impact they have on the ecology of the gut. We detected the presence of some symbionts (Oxymonads) that are almost exclusively found in the hindguts of wood-feeding termite and cockroach species (66). It is possible that these sequences represent a closely related symbiont lineage that may inhabit the hindgut of B. germanica, but additional studies are needed to explore this possibility. Interestingly, we also detected sequences associated with order Clevelandellida, and species within the genus Clevelandella have been described from wood-feeding cockroaches of the genus Panesthia (67). Their presence in wood feeders and in B. germanica calls into question whether these ciliates are parasites or symbionts. Finally, we detected sequences of other invertebrate parasites in the feces of field-collected B. germanica but not in lab-reared cockroaches, likely a representation of the local environment. Gregarina species are apicomplexan parasites, and infections have been described in colonies of B. germanica (68). The spore-forming N. blattellae is found in association with B. germanica Malpighian tubules (69). Infection initiates through oral uptake, as with other microbes, and then intracellular stages of infection are found in the Malpighian tubules, plasmodia are released into the tubule lumen, and mature pansporoblasts are transported into the gut and released with feces. It is interesting to note that this pathway also might expose the cockroach to infection with other microbes, as nephridiophagids create lesions that could allow gut bacteria to enter the hemocoel.

### Blattella germanica in the indoor environment.

Blattella germanica lives in close association with humans in indoor habitats (31). Several potentially pathogenic bacteria have been isolated from the gut and the exterior surface of cockroaches collected from hospitals (38, 40, 70), and bacteria carrying antibiotic resistance genes have been isolated from cockroaches collected in livestock operations (41, 42). Our results of congruence of the gut and fecal microbiomes indicate that the B. germanica gut can be colonized by potentially human-pathogenic bacteria, which can be dispersed in feces.

This study provides a comprehensive report that explored the diversity of the gut and fecal microbiota (both prokaryotic and eukaryotic) of cockroaches and examined the effect of geographic location on differentiation of the gut and fecal microbiomes. These results naturally lead to several pivotal questions on the effects of cockroaches on indoor microbiomes and cockroaches as vectors of potential human pathogens and antibiotic resistance genes in hospitals and animal farms. The German cockroach appears to be an excellent model to explore the mechanisms of coprophagy that ensure the establishment of a stable gut microbiota community.

## MATERIALS AND METHODS

### Lab-reared and field-collected cockroaches.

The lab colony was a strain of B. germanica (American Cyanamid = Orlando Normal) collected in a Florida apartment >70 years ago and reared on water and rodent chow (Purina 5001 rodent diet; PMI Nutrition International, St. Louis, MO) at 27 ± 1°C, 40 to 70% relative humidity, and a 12/12-h light/dark (L/D) photoperiod. Ten virgin females and males (5 and 10 days old, respectively) from the B. germanica lab colony were placed individually into 15-ml sterile centrifuge tubes, and their feces was collected for 2 days. Cockroaches were not provided with food or water during this period. After 48 h, cockroaches were separated into individual 1.5-ml tubes. The tubes with feces were stored at −30°C until DNA extraction.

Field-collected B. germanica cockroaches were from four different homes in Raleigh, NC, where their presence was reported by residents (North Carolina State University IRB#3796). Cockroaches were collected using a vacuum cleaner with a modified wand that trapped cockroaches, and they were immediately placed on ice and transported to the lab. Adult females and males were individually placed into 15-ml sterile centrifuge tubes within 2 h after collection, without food or water. After 48 h, the cockroaches were separated into individual 1.5-ml tubes, and the tubes with feces were stored in a −30°C freezer until DNA extraction.

### Gut dissections.

Cockroaches were surface sterilized using a modified protocol from Andrews (71). Briefly, the cockroaches were washed with 0.5% sodium hypochlorite and 70% ethanol and several washes with sterile water. After the last wash, the cockroaches were kept in 1% homogenization buffer (below) and placed on ice. The last wash was tested on culture plates and by PCR amplification (16S rRNA gene, see below) for contamination, using a 1-μl or 5-μl final wash solution as template per 20-μl reaction mixture. No growth was observed on culture plates, and no PCR amplicons were detected by electrophoresis on agarose gels. Dissections were carried out in a laminar flow hood with sterile equipment, and the alimentary canal and remaining body parts (here, carcass) were placed into separate 1.5-ml centrifuge tubes in 50 μl of sterile 1% homogenization buffer and stored at −30°C until DNA extraction.

### DNA extraction, quantitation, and PCR amplification.

Gut, carcass, whole-insect, and fecal samples were extracted separately using the DNeasy blood and tissue extraction kit (catalog no. 69506; Qiagen, Germantown, MD), per the manufacturer's protocol. Briefly, the samples were thoroughly homogenized with a motorized sterile pestle for ∼1 min until all the contents were thoroughly macerated, and 180 μl of ATL solution and 20 μl of proteinase K were added, vortexed thoroughly, and incubated for 60 min (gut, carcass, whole insect) or 30 min (feces) at 56°C. The rest of the protocol was performed according to the manufacturer's protocol. DNA was eluted in 100 μl sterile nucleic acid-free H_2_O. The genomic DNA of all samples was cleaned by ethanol precipitation. The concentration of extracted DNA was measured using a NanoDrop 1000 spectrophotometer (Thermo Scientific, Wilmington, DE). All the extracted samples were tested by amplifying the 16S rRNA gene using universal primers, 27F (5′-AGAGTTTGATCMTGGCTCAG-3′) and 1492R (5′-GGTTACCTTGTTACGACTT-3′), for PCR inhibitors (72). All the samples amplified and produced the proper size bands in subsequent electrophoresis on agarose gels. The DNA samples were stored at −20°C until further use.

### Quantification of 16S rRNA genes and Blattabacterium-specific genes.

Quantitative PCR was conducted to analyze the absolute abundances of total bacteria and Blattabacterium spp. in the whole-insect carcass sample. The previously published primers 338F (ACTCCTACGGGAGGCAGCAG) and 518R (ATTACCGCGGCTGCTGG) were used for total bacteria (73). However, previously published primers targeting the 16S rRNA gene of Blattabacterium spp. were not specific enough to amplify Blattabacterium alone in the whole-insect samples. Therefore, a new set of primers was designed for this study targeting the penicillin-binding protein (PBP) gene in the Blattabacterium genome. These novel primers, Blattabacterium-PBPF (GGCTGGAAAAACTGGAACGG) and Blattabacterium-PBPR (CAATAGGTCCAGCCCAACGA), amplify a 173-bp region of the PBP gene in the Blattabacterium chromosome. The new primers were validated against positive (genomic DNA [gDNA] from B. germanica egg case and whole insects) and negative (Escherichia coli) controls for specificity and confirmed by Sanger sequencing. The amplicons from the B. germanica egg case (for Blattabacterium-PBP) and E. coli (for 16S rRNA) from respective PCRs were cloned using the pGEM-T cloning kit (catalog no. A1360; Promega, Madison, WI, USA). The plasmid DNA from the positively identified clones (amplified by M13 primers and sequence identity confirmed by Sanger sequencing) was used for making standard curves for qPCR assays. Quantitative PCR assays were carried out on a CFX384 Touch real-time PCR detection system (Bio-Rad, USA) in a reaction mixture of 15 μl containing 0.5 μM (each) gene-specific primers, 7.5 μl of SsoFast EvaGreen supermix (Bio-Rad, USA), 1.5 μl of DNA template, and nuclease-free water to make up the 15-μl volume. The qPCR conditions for the 16S rRNA gene and PBP gene were similar except the annealing temperatures of 55°C and 59°C for the two gene targets, respectively. The real-time PCR conditions were 3 min at 95°C for initial activation, 40 cycles of 15 s at 95°C for denaturation, 15 s at 55°C and 59°C for 16S rRNA and Blattabacterium-PBP, respectively, for annealing, and 30 s at 72°C, followed by a melt curve from 65°C to 95°C, with an increment of 0.5°C. Positive (gDNA from E. coli and B. germanica egg case for total bacteria and Blattabacterium spp., respectively) and negative (no DNA) controls were included in all qPCR runs. The total copy numbers of 16S rRNA and PBP genes were calculated by respective standard curves, and the percent abundance of Blattabacterium spp. in qPCR assay was calculated as follows:

%abundance of Blattabacterium in qPCR=(PBP copy number/16S rRNA copy number)×100

We used Pearson's correlation to investigate the relationship between qPCR and NGS data to analyze the abundance of Blattabacterium species.

### NGS library preparation.

The amplification protocols and primers were done as per the Earth Microbiome Project (http://www.earthmicrobiome.org/). Genomic DNA was amplified using 16S rRNA gene primers 515F (GTGCCAGCMGCCGCGGTAA) and 806R (AGTCAGTCAGCCGGACTACHVGGGTWTCTAAT), and 18S rRNA gene primers 1391F (GTACACACCGCCCGTC) and EukBr (TGATCCTTCTGCAGGTTCACCTAC) (74) and the mammal blocking primer designed as described by Vestheim and Jarman (75). Unique Golay barcodes used in 16S and 18S rRNA gene analyses for multiplexing were linked to the reverse primer and are presented in Tables S2 and S3, respectively, in the supplemental material. Each sample was amplified in triplicate along with extraction and PCR controls. Once the negative controls were confirmed free of contamination, the 3 replicates of each sample were combined and purified using AMPure beads (catalog no. A63881; Agilent Technologies, Santa Clara, CA), per the manufacturer's protocol. The samples were tested on Agilent 2200 TapeStation D1000 ScreenTapes (Agilent Technologies) for the presence of the correct size bands and quantified using the Quant-iT double-stranded DNA (dsDNA) assay kit (catalog no. Q33120; Invitrogen-Thermo Fisher, Waltham, MA). Quantified libraries were individually normalized to 4 nM, and equal amounts of each 4 nM dilution were pooled for sequencing.

### 16S rRNA gene and 18S rRNA gene sequencing.

All sequencing was performed at the NYU Center for Genomics and Systems Biology Genomics Core facility using the Illumina MiSeq platform (San Diego, CA). The 16S rRNA gene amplicon libraries from 105 samples were sequenced in three runs using a 500-cycle V2 kit and 2 × 250-run configuration. The 18S rRNA gene amplicon libraries from 63 samples were sequenced in two runs using a 300-cycle version 2 kit and a 2 × 100-run configuration.

### 16S rRNA gene sequence analysis.

Quality filtering and analysis of the 16S rRNA gene sequence data were performed with QIIME version 1.9.1 (76). Briefly, the reads from each run were joined using fastq-join (77), with a minimum overlap of 5 bp and allowing a 15% error rate in the overlapping area. Joined reads from each run were demultiplexed (split_libraries_fastq.py, -q 19 –r 5 –p 0.70), and the reads from the three runs were merged after truncating the reverse primer. The demultiplexed sequences were taken through chimera checking and binned into operational taxonomic units (OTUs) using an open-reference OTU picking strategy at 97% using the USEARCH pipeline (USEARCH version 8.0.1) (78). Taxonomic assignment of OTUs was done using BLAST against the Greengenes reference database version 13.8 (79). OTUs that were observed <2 times, i.e., singletons and OTUs that were identified as chloroplasts, were filtered from the data set.

### Bioinformatics of 16S rRNA sequences.

Diversity analysis was performed on a rarefied OTU table at a sampling depth of 50,000 sequences/sample. The beta diversity of the bacterial communities was calculated using weighted and unweighted UniFrac methods (80). Principal-coordinate analyses (PCoA) were conducted, and diversity patterns were visualized using Emperor tools. Statistical comparisons between treatments were conducted using adonis (81) and analysis of similarity (ANOSIM) (82). Alpha diversity was calculated using phylogenetic distance, nonphylogenetic metrics, including Shannon entropy, and Chao1, and observed species and nonparametric Kruskal-Wallis tests were used for statistical analysis. The PD_Whole tree, Shannon diversity index, Good's coverage index, Chao1 index (83), and number of observed species were used as metrics to plot alpha-rarefaction curves. Relative abundances of bacterial orders from different treatments (gut versus carcass, male versus female, lab versus field) were compared using Mann-Whitney U tests, followed by Benjamini-Hochberg correction (*q* < 0.05) in the QIIME pipeline.

### 18S rRNA gene sequence analysis.

Raw reads were trimmed of adapter sequences using Trimmomatic version 036 (84), and paired-end reads were joined within the QIIME 1.9.0 (76) pipeline using fastq-join (85), with a minimum overlap of 5 bp and allowing a 20% error rate in the overlapping area. Joined reads were demultiplexed and quality filtered (split_libraries_fastq.py, -q 19 –r 5 –p 0.70). Demultiplexed reads were subjected to *de novo* chimera checking and removal of singletons, and they were clustered into OTUs at 98% identity following the UPARSE pipeline (USEARCH version 8.0.1) (86). Taxonomy was assigned to representative OTU sequences using BLAST (87) within QIIME, first against a curated SILVA database (88) and subsequently with the QIIME-formatted SILVA 111 database clustered at 99% identity (89). OTUs with no significant hits (<90% identity) after both rounds of taxonomic assignment were labeled as “unidentified.” OTUs were then filtered to remove non-18S rRNA gene sequences (bacterial and archaeal OTUs), host sequences (B. germanica), and low-abundance OTUs making up <0.001% of reads in the total data set, as recommended for Illumina sequencing data (90).

### Bioinformatics of 18S rRNA sequences.

Eukaryotic OTU tables were rarefied to 5,000 sequences per sample prior to alpha-diversity analysis. Alpha diversity was calculated on rarefied OTU tables using the Shannon diversity index within the QIIME pipeline. Beta diversity and ordinations were calculated by nonmetric multidimensional scaling (NMDS) using the Bray-Curtis dissimilarity in R with the vegan package version 2.4-3 (metaMDS, k = 2, trymax = 200). The association of community composition with metadata factors was assessed by the adonis function in R, with 10,000 permutations, and adjusted using the false-discovery rate (FDR) correction for multiple testing. Relative abundance analyses were conducted on sum-normalized OTU tables. Multivariate association tests for taxa that were differentially abundant between field-collected cockroaches were performed using MaAsLin, with default parameters (91).

### Accession number(s).

The sequences from this study were deposited in the NCBI SRA database under BioProject no. PRJNA415481 and Biosample accession numbers SAMN07823957 (16S rRNA gene) and SAMN07823958 (18S rRNA gene).

## Supplementary Material

Supplemental file 1

Supplemental file 2

Supplemental file 3

Supplemental file 4

Supplemental file 5

Supplemental file 6

Supplemental file 7

Supplemental file 8
