# Supplementary material for: Overlapping Community Compositions of Gut and Fecal Microbiomes in Lab-Reared and Field-Collected German Cockroaches
Source: Appl Environ Microbiol. 2018 Aug 17;84(17):e01037-18. doi: 10.1128/AEM.01037-18 (PMC6102980; doi:10.1128/AEM.01037-18)
Supplement: Supplemental file 1 [file zam017188703s1.pdf]

Supplementary figure S1: Rarefaction curves generated from non-phylogenetic and phylogenetic metrices (a) observed species, (b) Chao1 (c) Shannon diversity (d) PD_whole tree and e) Good's coverage index by location.

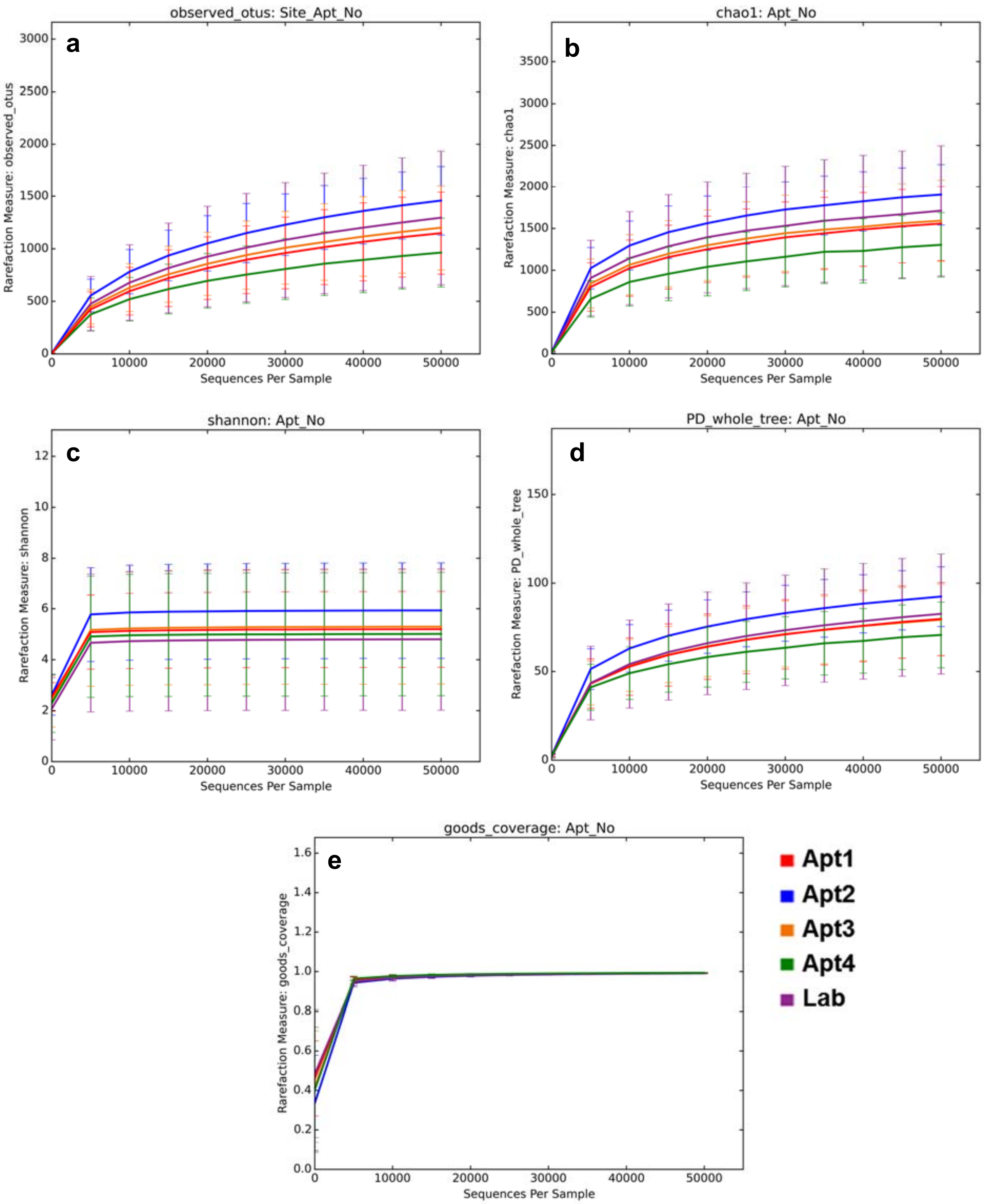

Supplementary figure S2: Taxonomic assignment of 16S rRNA gene of lab-reared and field-collected (Apt = apartment) male and female German cockroaches (whole insect samples) at the phylum level.

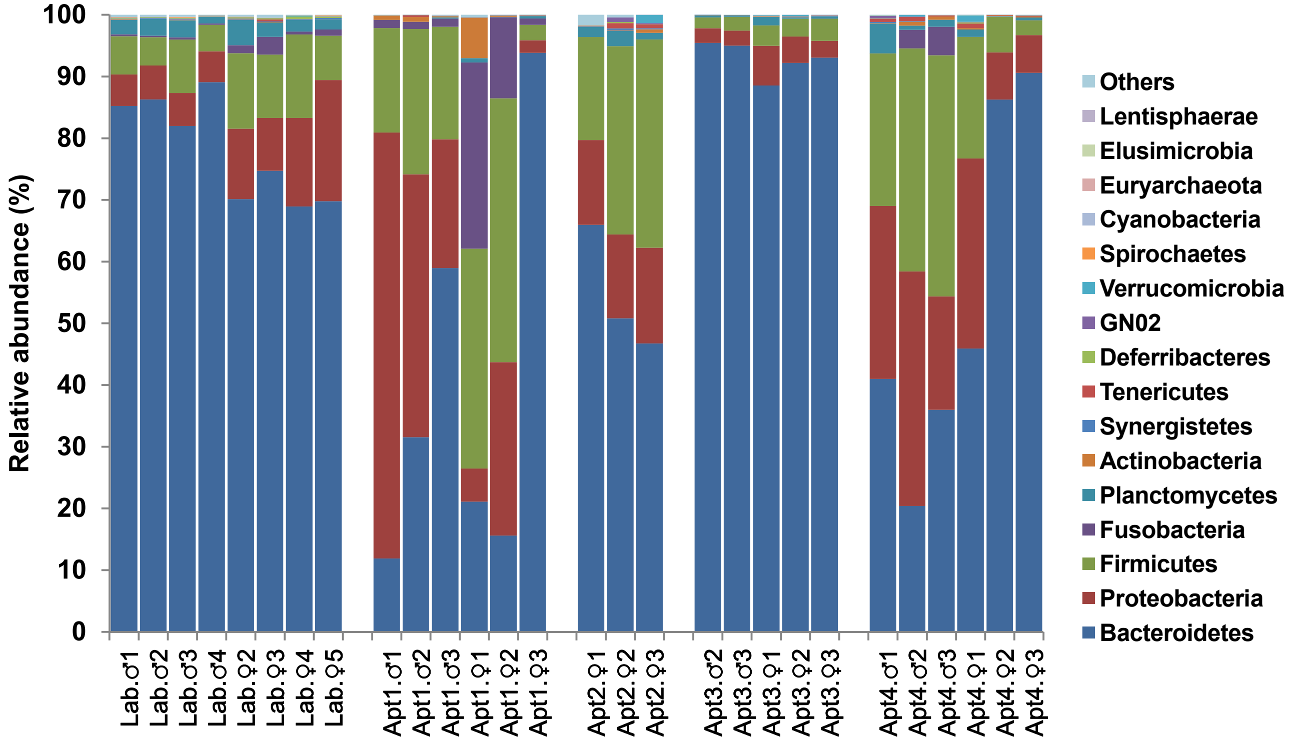

Supplementary figure S3: a) The distribution of bacterial taxa at the family level in whole body samples of male and female *B. germanica* cockroaches from lab-reared and field-collected cockroaches presented by location. Bars depict the mean relative abundance value of independent replicates (Lab: M=4, F=4; Apt1: M=3, F=3; Apt2: F=3; Apt3: M=2, F=3; Apt4: M=3, F=3). b) Principal coordinate analysis depicting differences in bacterial community composition among independent replicates of lab colony whole German cockroach samples (males (Δ), females (O)) based on the Bray-Curtis dissimilarity metrics. c) Column graphs depicting the mean relative abundance of Blattabacteriaceae and Bacteroidaceae in males and females in lab colony d) Blattabacteriaceae among field populations.

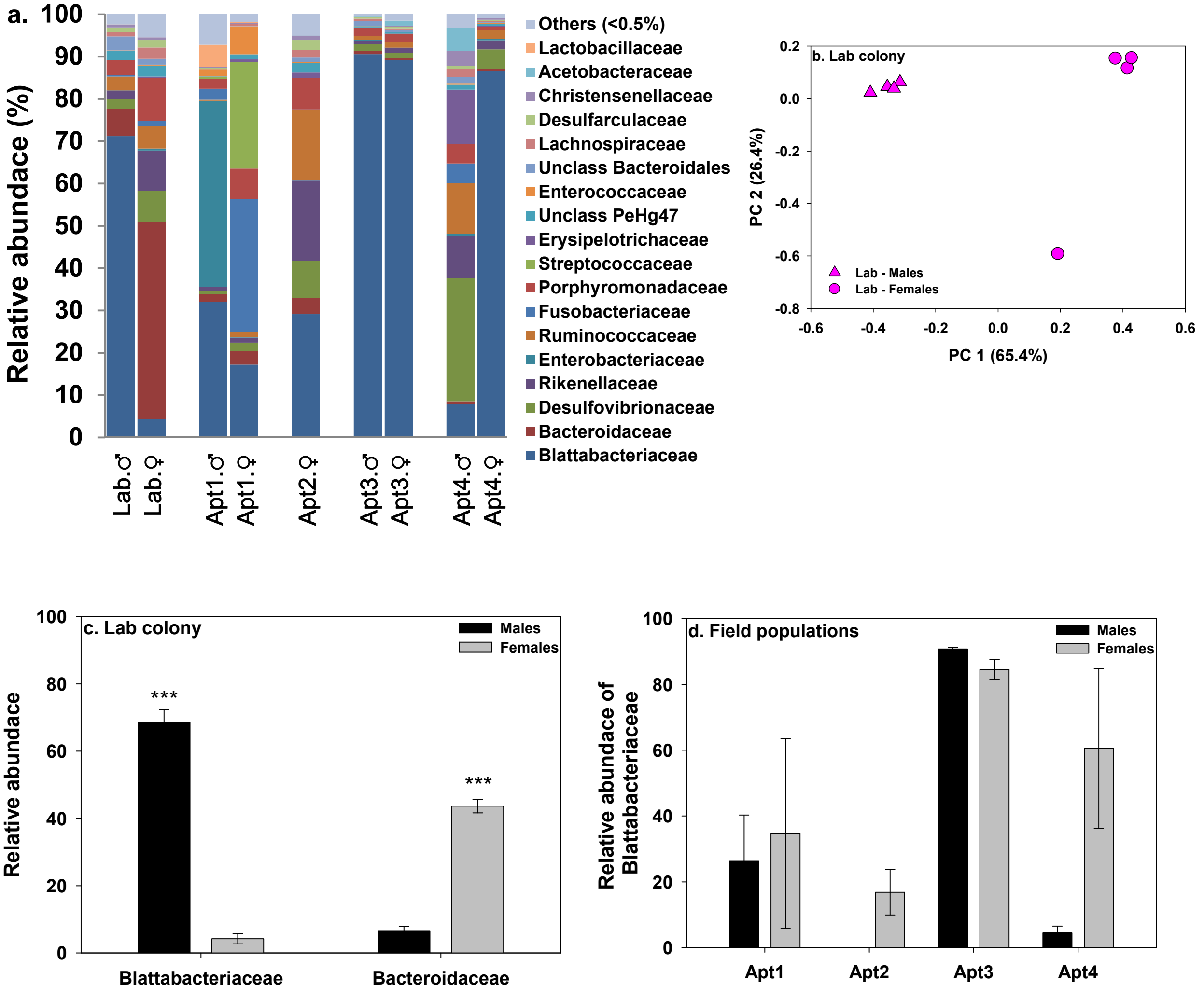

Supplementary figure S4: Principal coordinate analysis depicting differences in bacterial community composition among independent replicates of field-collected whole German cockroach samples (males (Δ), females (O)) based on the Bray-Curtis dissimilarity metrics. Percentage variation explained by each component is indicated on the axis.

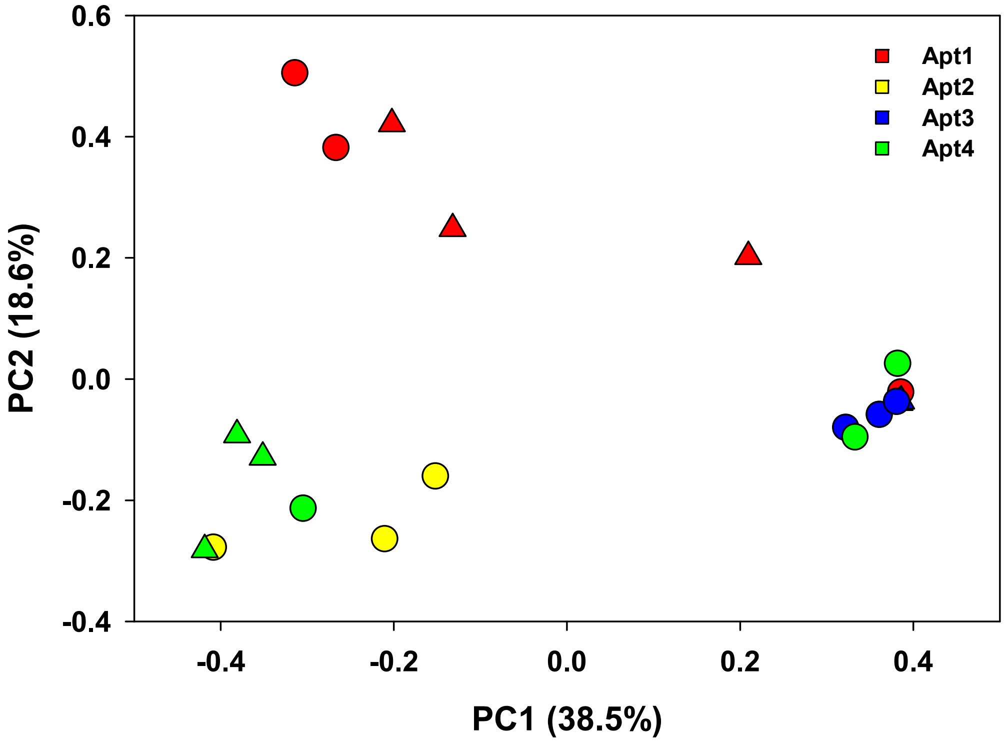

Supplementary figure S5: Boxplots showing the alpha diversities among the gut and feces of *B. germanica* cockroach samples by location. Boxplots show a) observed species, b) Shannon diversity indices and c) Phylogenetic diversity of gut and fecal samples. For each group, the bars delineate the median and 'X' defines the mean, the hinges represent the lower and upper quartiles, the whiskers extend to the most extreme values (no more than 1.5 times the interquartile range from the box), and outliers are plotted in circles, if present. Sample sizes for gut (G) and feces (FM) are Lab: G=9, FM=9, Apt1: G=8, FM=7, Apt2: G=8, FM=8, Apt3: G=8, FM=8.

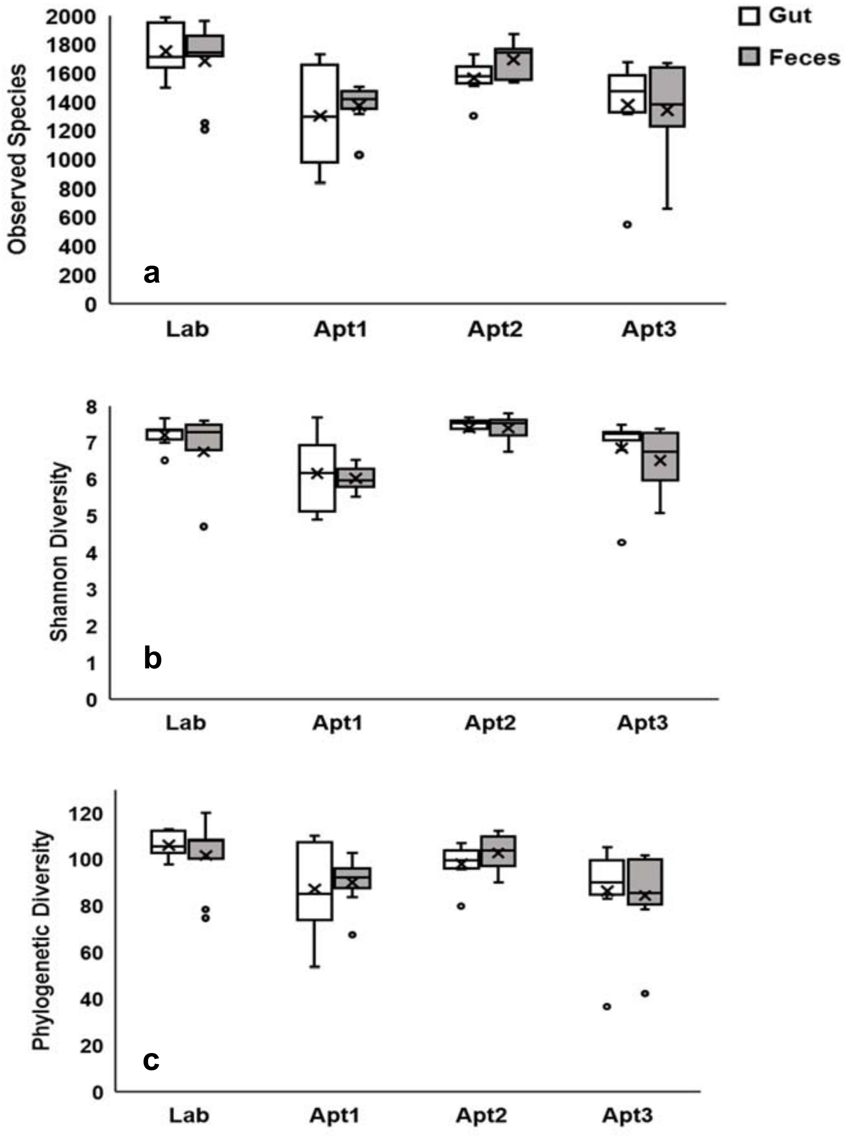

Supplementary figure S6: Taxonomic assignment of 16S rRNA gene of gut and feces (FM) samples of male and female German cockroaches from field (a) Lab (b) Apartment 1 (c) Apartment 2 (d) Apartment 3 at the family level, excluding *Blattabacterium*.

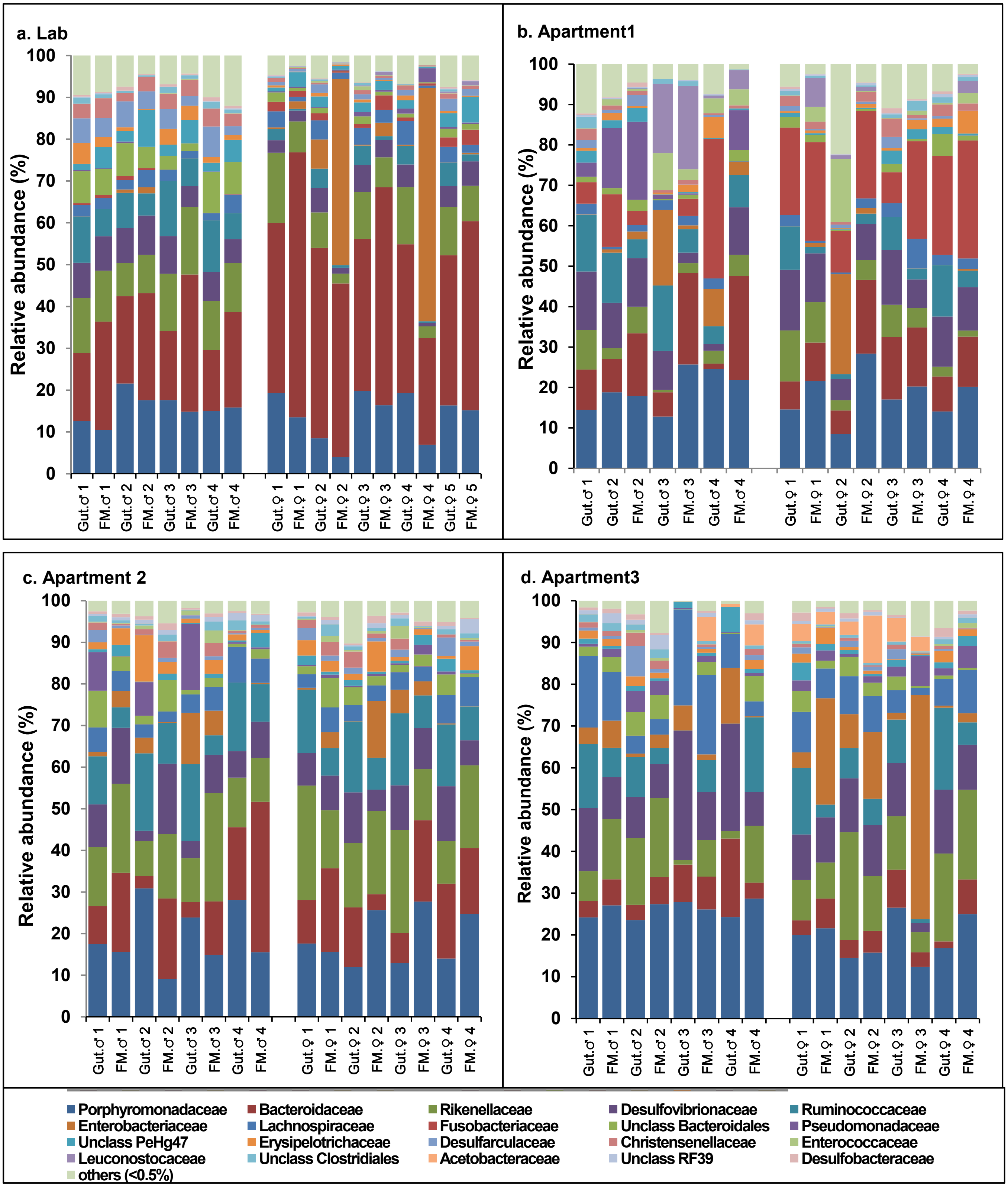

Supplementary figure S7: a) Relation between the qPCR and NGS data in the relative abundance of *Blattabacterium* in whole-insects, whole-gut, feces and carcass samples of Lab-reared and field-collected samples (n=99). b) The log copy number of 16S rRNA and *Blattabacterium* specific Penicillin Binding Protein (PBP) genes in carcass samples of males and females of Lab-reared and Field-collected cockroaches. Sample sizes for Lab: Males (M) = 4, Females (F) = 5, Apt1: M = 4, F = 4, Apt2: M = 4, F = 4, Apt3: M = 4, F = 4.

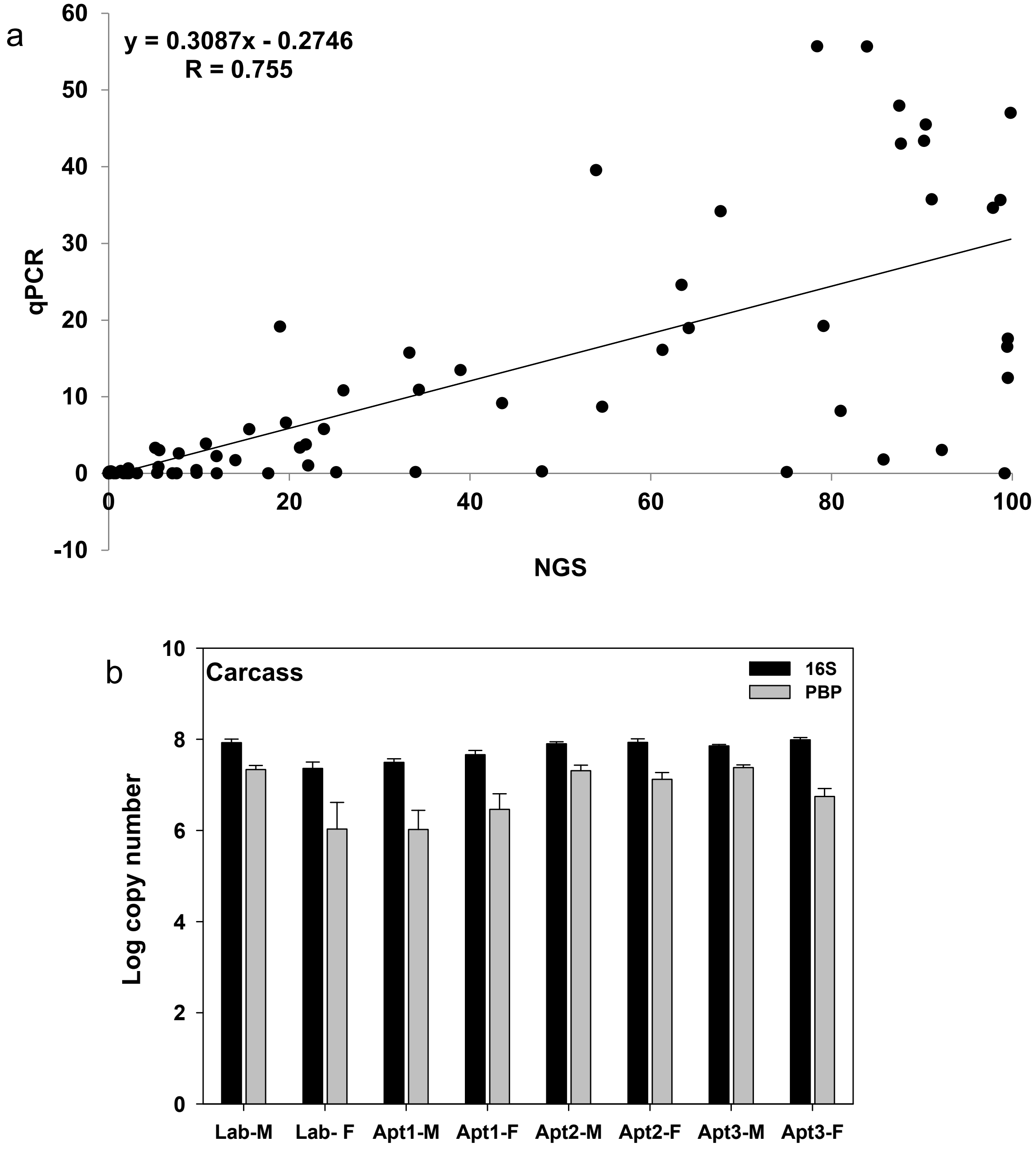

Supplementary Table S1: Sample details of lab-reared and field-collected *Blattella germanica* included in the study.

Excel sheet Attached

Supplementary Table S2: Metadata and Golay barcodes used for 16S sequencing

Excel sheet Attached

Supplementary Table S3: Metadata and Golay barcodes used for 18S sequencing

Excel sheet Attached

Supplementary Table S4: Summary statistics table for 16S data showing total, average number of reads, total OTUs and the number of reads by sample.

Excel sheet Attached

Supplementary Table S5: Relative abundance of bacterial species (L6) observed in the gut of lab-reared and field-collected cockroaches after excluding the prominent endosymbiont *Blattabacterium*.

Excel sheet Attached

Supplementary Table S6: Core microbiota observed in 100% and 95% of *B. germanica* samples.

Excel sheet Attached

Supplementary Table S7: MaAsLin analysis for 18S sequencing.

Excel sheet Attached
